# Diversity of Gut Microbiota and Bifidobacterial Community of Chinese Subjects of Different Ages and from Different Regions

**DOI:** 10.3390/microorganisms8081108

**Published:** 2020-07-24

**Authors:** Bo Yang, Shuang Yan, Yang Chen, R. Paul Ross, Catherine Stanton, Jianxin Zhao, Hao Zhang, Wei Chen

**Affiliations:** 1 State Key Laboratory of Food Science and Technology, Jiangnan University, Wuxi 214122, China; bo.yang@jiangnan.edu.cn (B.Y.); yans678@163.com (S.Y.); chenyiquen@yeah.net (Y.C.); zhaojianxin@jiangnan.edu.cn (J.Z.); zhanghao61@jiangnan.edu.cn (H.Z.); 2 School of Food Science and Technology, Jiangnan University, Wuxi 214122, China; 3 International Joint Research Center for Probiotics & Gut Health, Jiangnan University, Wuxi 214122, China; catherine.stanton@teagasc.ie; 4 APC Microbiome Ireland, University College Cork, T12 K8AF Cork, Ireland; 5 Teagasc Food Research Centre, Moorepark, Fermoy, P61 C996 Co. Cork, Ireland; 6 National Engineering Research Center for Functional Food, Jiangnan University, Wuxi 214122, China; 7 Wuxi Translational Medicine Research Center, Jiangsu Translational Medicine Research Institute Wuxi Branch, Wuxi 214122, China; 8 Beijing Innovation Center of Food Nutrition and Human Health, Beijing Technology and Business University (BTBU), Beijing 102488, China

**Keywords:** Chinese subjects, gut microbiota, bifidobacterial community, diversity, region, age

## Abstract

Gut microbiota composition and functionality are closely linked to host health. In this study, the fecal microbiota and bifidobacterial communities of 111 healthy volunteers from four regions of China of varying age profiles (Child, 1–5 years; Young, 18–50 years; Elder, 60–80 years; Longevity, ≥90 years) were investigated via high-throughput sequencing. Canonical analysis revealed that the gut microbiota, as well as bifidobacteria profiles of the subjects, clustered according to their regions and age. Eight genera were shared among all subjects, however, certain genera distributed differently in subjects grouped by region and age. *Faecalibacterium* was enriched in samples from Zhongxiang, unclassified *Ruminococcaceae* and *Christensenellaceae* were enriched in the Longevity group, and *Bifidobacterium* was enriched in Child. Within *Bifidobacterium*, *B. longum* was the most abundant species in almost all samples except for Child, in which *B. pseudocatenulatum* was the most abundant. Additionally, the abundances of *B. adolescentis* and *B. dentium* were lower in Child. In conclusion, our results suggest that geography and age affect the structure of the gut microbiota, as well as *Bifidobacterium* composition, and this variation may greatly associate with the metabolic and immune changes that occur during the process of aging.

## 1. Introduction

The human gut microbiota is an extremely complex ecosystem with a biomass of 0.15 kg dry weight [1] and comprises trillions of microorganisms, which are intricately linked to human physiology and health [2]. Numerous commensal bacteria co-evolve and interact with the mammalian host during its lifetime [3]. The gut microbiome plays a significant role in the development and regulation of the immune system and in the development of gut tissue along with serving a multitude of other functions including colonization resistance and energy regulation, as examples [4,5,6,7,8]. Thus, disruptions and imbalances to the gut microbiome are connected to several diseases [9,10,11,12]. In recent years, research has focused on unraveling the composition of the microbiota, such as the National Human Microbiome Program (HMP) [13], Metagenomics of the Human Intestinal Tract (MetaHIT) [14,15] and many other projects [16,17]. Understanding the composition and functionality of the gut microbiome in health and disease offers potential targets for the diagnosis, therapy and prevention of some diseases, as well as promotion of personalized medicine based on nutrition, microorganisms and drugs.

The gut microbiota in healthy people is influenced by multiple factors, such as age, environment and diet. The physiological changes to the gastrointestinal tract induced by ageing inevitably influence the structure and functioning of the gut microbiota. Indeed, the early infant gut microbiota is represented by low biodiversity which increases rapidly in the first 2–5 years until it resembles that of an adult [18], the adolescent and adult microbiota is relatively stable while the elderly period is defined by a decline in microbial diversity [16,19].

*Bifidobacterium*, one of the dominant bacteria in the human gut microbiota, especially in breast-fed infants, was first discovered in 1899 by Tissier [20]. Bifdobacteria colonize the large intestine of full-term, healthy and breast-fed infants during the first weeks owing largely to their capacity to utilize human milk oligosaccharides in breast milk. Certain *Bifidobacterium* strains have proven health benefits with disease alleviating effects [21,22,23,24].

With the development of sequencing technologies, culture-independent approaches based on next-generation sequencing have been widely used as powerful tools to detect the gut microbiota or *bifidobacterial* community of individuals [25]. This has been largely based on marker gene metagenomics, particularly 16S rRNA gene amplicon sequencing, which is widely used for bacterial compositional analysis. However, the resolvability of 16S rRNA gene sequences is limited and cannot distinguish closely related bacterial species [26,27]. In view of this, our laboratory has developed a high-throughput sequencing method for the detection of *Bifidobacterium* species based on the groEL gene, which can assess the diversity of *bifidobacterial* composition to species-level [28]. Using both approaches (16S rRNA gene sequencing and groEL gene sequencing) in this study, we assessed the influence of geography and age on the structure of the gut microbiota and composition of *Bifidobacterium*. In total, we collected 111 fecal samples from four regions in China, which could be divided into four groups according to age; Child (1–5 years), Young (18–50 years), Elder (60–80 years), and Longevity (≥90 years). By using the Illumina MiSeq sequencing technology, we uncovered the properties of the gut microbiota and *Bifidobacterium* composition in fecal samples of individuals from different regions and of different ages.

## 2. Materials and Methods 

### 2.1. Subject Recruitment and Fecal Sample Collection

This study was approved by the Ethics Committee of Jiangnan University, China (SYXK 2012-0002, 15 February 2015). All the fecal samples from healthy persons were for public health purposes and these were the only human materials used in the present study. Written informed consent was obtained from the participants or their legal guardians. Health questionnaires were conducted before sampling and no human experiments were involved. The collection of fecal samples had no risk of predictable harm or discomfort to the participants. A total of 111 volunteers were recruited from four regions in China with different ages (Table S1), and the identity and age of each volunteer was confirmed via checking their household registers. None of these volunteers had gastrointestinal tract disorders or had taken antibiotics for at least three months before sampling. Fresh fecal samples were collected in the early morning before breakfast in a cooler with ice packs and transferred to the laboratory within 24 h and stored at −80 °C. Samples were grouped according to region and age, respectively; the number of volunteers from each region was as follows: Zhongxiang (21, 15 male and 6 female), Bo’ai (37, 15 male and 22 female), Chengmai (32, 22 male and 10 female) and Rugao (21, 6 male and 15 female); samples were grouped by age: Child (1–5 years), Young (18–50 years), Elder (60–80 years) and Longevity (≥90 years). 

### 2.2. DNA Extraction, PCR Amplification and High-Throughput Sequencing

The total DNA from fecal samples was extracted using FastDNA Spin Kit for Feces (MP Biomedicals, Santa Ana, CA, USA) according to the manual. The V4 region of the 16S rRNA gene was amplified with a universal bacterial primer pair: 520F (5′-AYT GGG YDT AAA GNG-3′) and 802R (5′-TAC NVG GGT ATC TAA TCC-3′) containing a seven-base barcode, and the PCR amplification procedures were performed as described previously [29]. The *groEL* gene was amplified with a *Bifidobacterium*-specific primer pair: 308F (5′-TCC GAT TAC GAY CGY GAG AAG CT-3′) and 806R (5′-CSG CYT CGG TSG TCA GGA ACA G--3′) containing a seven-base barcode, and the PCR amplification procedures were performed as described previously [28]. The PCR products were purified using QIAquick Gel Extraction Kit (Qiagen GmbH, Hilden, Germany) and quantified with Qubit™ 4 Fluorometer (Life Technologies Corporation, Carlsbad, CA, USA). Libraries were prepared with TruSeq Nano DNA LT Kit (Illumina, San Diego, CA, USA) and sequenced with the Miseq Reagent Kit v3 (600 cycles-PE, Illumina) on the Illumina MiSeq platform according to manufacturer’s instructions.

### 2.3. Bioinformatics Analysis

Raw sequences were assembled using SeqPrep (https://github.com/jstjohn/SeqPrep) in a QIIME package (Quantitative Insights into Microbial Ecology, v1.9.1) with default parameters [30]. Reads that could not be assembled were discarded. Sequences with a fraction of consecutive high-quality base calls (phred score > 29) lower than 75%, containing ambiguous bases, or lacking a perfect match to given barcodes were removed. When a sequence had more than three consecutive low quality base calls (phred score < 30), it was truncated. A customized script was applied to extract and merge abundance data at different taxonomic levels. High-quality reads were clustered into operational taxonomic units (OTUs) for further taxonomic analysis. Representative sequences from each cluster were aligned with the PyNAST aligner to the Greengenes core set, and then a de novo taxonomic tree was constructed using FastTree v2.1.3 [31]. The taxonomic abundance of each sample was calculated with Ribosomal Database Project (RDP) classifier v2.10.2 trained with 16S rRNA training set No. 14 using a bootstrap cutoff of 50% [32]. Subsequently, a gene copy number adjustment for 16S rRNA sequences was performed. The 16S rRNA copy number data are provided by rrnDB website (https://rrndb.umms.med.umich.edu/) [33]. The bifidobacterial community profiles were carried out as previously described [28]. Chao1, Shannon and Simpson indexes were analyzed to estimate the alpha diversity, principal coordinates analysis (PCoA) was analyzed to estimate the beta diversity, and these analyses were performed by QIIME; canonical analysis of principle coordinates (CAP) and permutational multivariate analysis of variance (PERMANOVA) were performed to evaluate the difference of gut microbiota structure across the cohorts by R-package vegan [34,35]. Linear discriminant analysis effect size (LEfSe) was used to identify the signature microbes that best differentiate samples grouped by region or age (Wilcoxon rank–sum test, α < 0.05, log LDA >3) [36]. Phylogenetic Investigation of Communities by Reconstruction of Unobserved States (PICRUSt) was used to obtain a deeper insight into different pathways, based on Kyoto Encyclopedia of Genes and Genomes (KEGG) orthology [37].

### 2.4. Statistical Analysis

Statistical analysis was conducted with SPSS 20.0 and GraphPad Prism 7 software. Shapiro–Wilk normality test was used to verify the normality of distribution for the values. Independent Kruskal–Wallis test of one-way ANOVA or Tukey’s test of one-way ANOVA was performed to determine the significant differences among groups depending on the result of normality test. The results were expressed as median (minimum, maximum). Significant differences were defined as *p* < 0.05.

## 3. Results

### 3.1. Alpha-Diversity of Gut Microbiota of Subjects from Different Regions and across Age Groups

**Figure 1:**
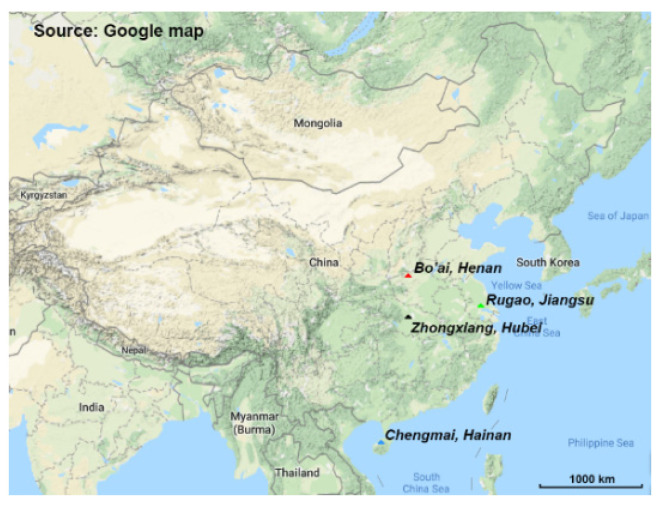
Sampling sites in this study. The four sampling sites are represented by colored triangles on the map. Red: Bo’ai (Henan Province); Green: Rugao (Jiangsu Province); Black: Zhongxiang (Hubei); Blue: Chengmai (Hainan Province).

**Figure 2:**
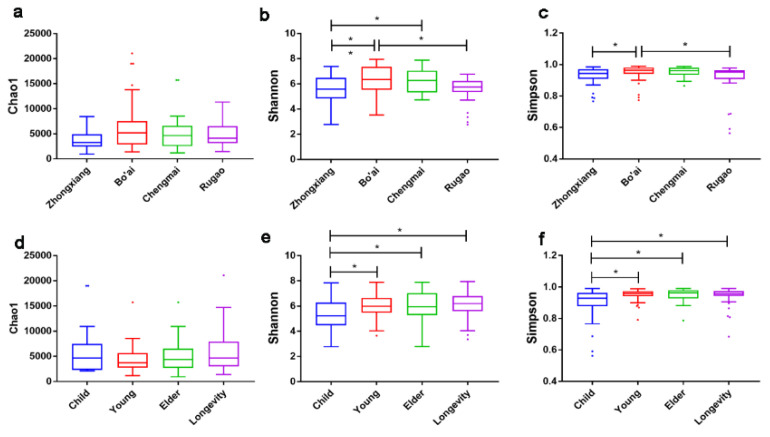
Alpha-diversity of gut microbiota among different regions and ages. Boxes represent the interquartile range (IQR) between the first and third quartiles, and the lines inside the boxes represent the median. Whiskers denote the lowest and highest values within 1.5× IQR from the first and third quartiles, respectively. The colored points represent the values with distance from the median exceeding 1.5× IQR. (**a**–**c**) Chao1, Shannon and Simpson indexes of each region. (**d**–**f**) Chao1, Shannon and Simpson indexes of each age. * indicates that the difference is significant, *p* < 0.05.

In total, 111 volunteers were recruited from four regions in China (Figure 1) with different age profiles (Table S1). A total of 4,913,866 high-quality 16S rRNA gene sequences were obtained from all the samples, and the average sequence number was 29,424 for each sample. Microbial diversity was analyzed through Chao1, Shannon and Simpson indexes (Figure 2), in which the Shannon index revealed significant differences across samples from different locations and with different ages (Figure 2b,e). Samples from Bo’ai had a significantly higher Shannon index than those from Zhongxiang and Rugao, indicating that the diversity of gut microbiota in subjects from Bo’ai was higher. The Shannon index of Chengmai was similar to that of Bo’ai but higher than that of Zhongxiang and Rugao, although the difference between Chengmai and Rugao was not significant. The Simpson index of Bo’ai was also higher than that of Zhongxiang and Rugao. Among samples of different ages, the Shannon and Simpson indexes of Child were significantly lower than that of others. There was no significant difference among Chao1 indexes of each group.

### 3.2. Beta-Diversity of Gut Microbiota of Subjects from Different Regions and across Age Groups

**Figure 3:**
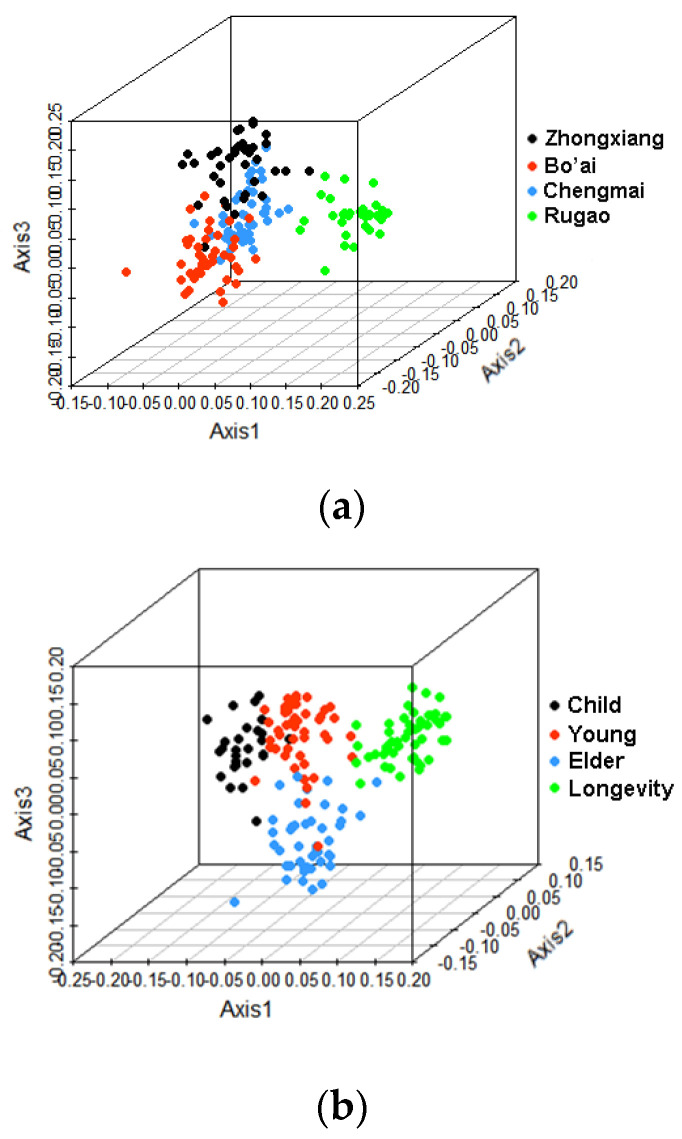
Canonical analysis of principal coordinates (CAP) based on high-throughput sequencing data of the V4 region of the 16S rRNA gene. (**a**) CAP of gut microbiota of different region groups. (**b**) CAP of gut microbiota of different age groups.

Principal coordinates analysis (PCoA) of weighted UniFrac distances was analyzed based on the relative abundances of OTUs to compare the overall structure of the gut microbiota. The results of PCoA revealed no obvious separation of samples from different regions or with different ages (Figure S1), however, the results of the canonical analysis of principal coordinates (CAP) indicated that the samples from different regions and with different ages separated from each other (Figure 3), which were confirmed by PERMANOVA test (Tables S2 and S3), suggesting that bacterial compositions differed within each group.

### 3.3. Composition of Gut Microbes of Subjects from Different Regions and Age Profiles

**Figure 4:**
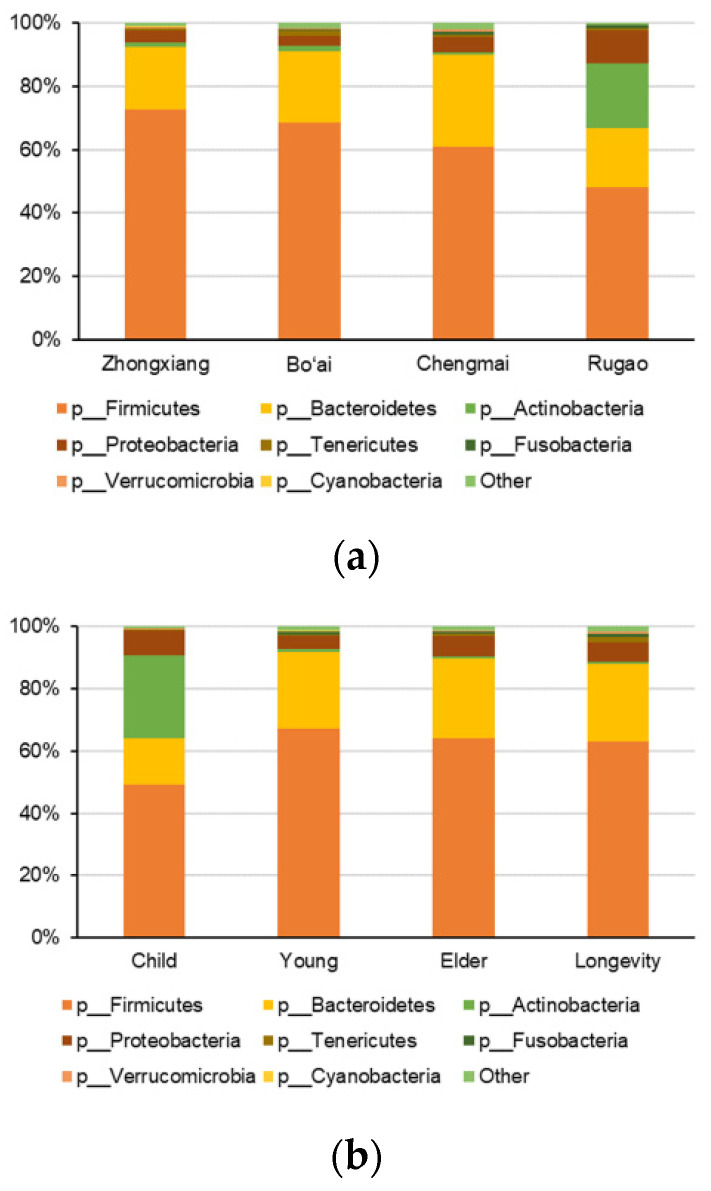
The composition of gut microbiota at phylum level within subjects from different regions (**a**) and across age groups (**b**).

The overall microbiota profiles at phylum level are presented in Figure 4. Eight phyla were detected with relative abundances of more than 0.1% in all the samples. The dominant phyla were *Firmicutes*, *Bacteroidetes*, *Proteobacteria* and *Actinobacteria*, where *Firmicutes* was the dominant phylum across all groups. Further, the four phyla accounted for more than 48% of the total microbiota. Samples from Rugao had higher relative abundances of *Actinobacteria* and *Proteobacteria*, but without significant differences (Figure 4a). However, *Actinobacteria* differed across the different age groups, whereby the relative abundance of *Actinobacteria* in Child was significantly higher than that of individuals in Elder and Longevity groups (*p* = 0.012 and 0.004, respectively), but the difference between Child and Young was not significant (Figure 4b).

**Figure 5:**
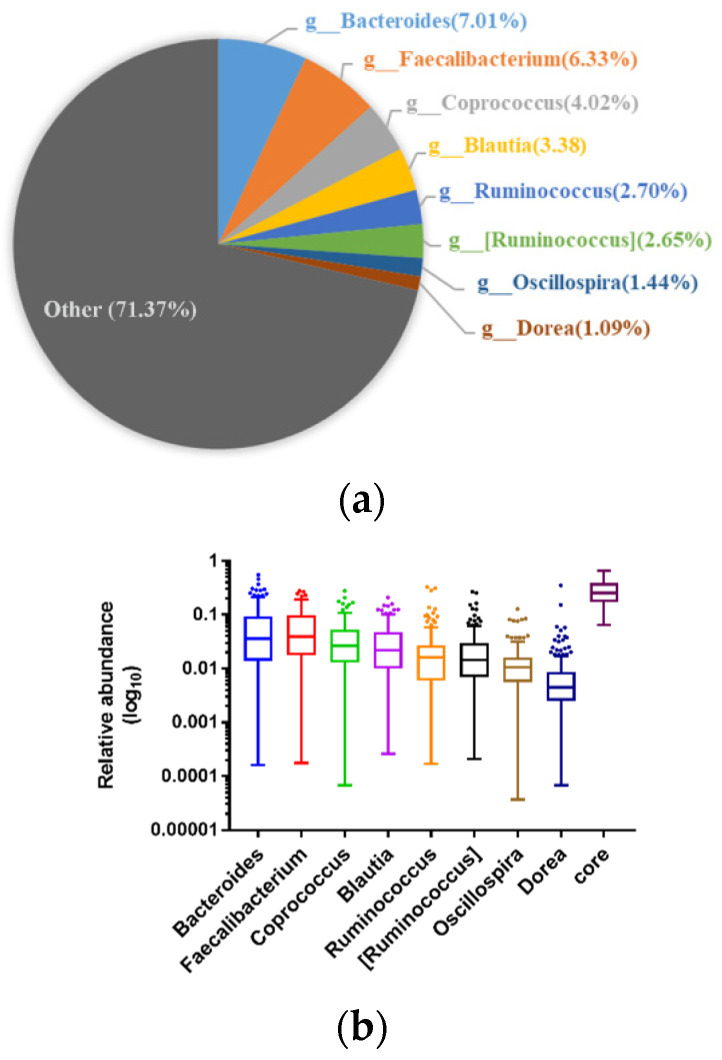
The core genera shared by all the samples. (**a**) represents the average proportion of each core genus within the whole sequences. (**b**) represents the distribution of the relative abundance of each genus and their collection. Boxes show the interquartile range (IQR) between the first and third quartiles, and the lines inside boxes represent the median. Whiskers denote the lowest and highest values within 1.5× IQR from the first and third quartiles, respectively. The colored points present the values with distance from the median exceeding 1.5 times of IQR.

At genus level, a total of 407 genera were detected in which eight genera existed in all the samples composing a genus-level phylogenetic core and accounting for 28.63% of the total microbiota (Figure 5). 

**Figure 6:**
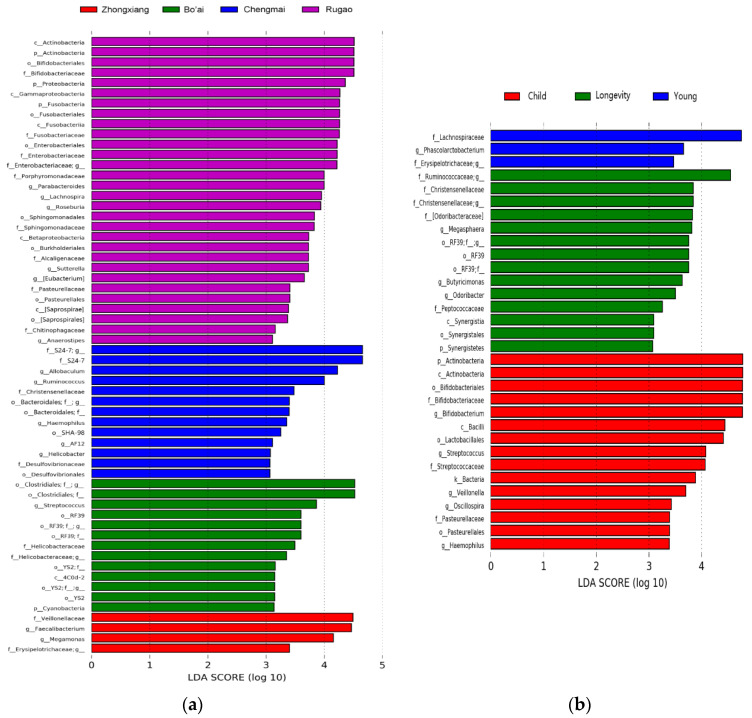

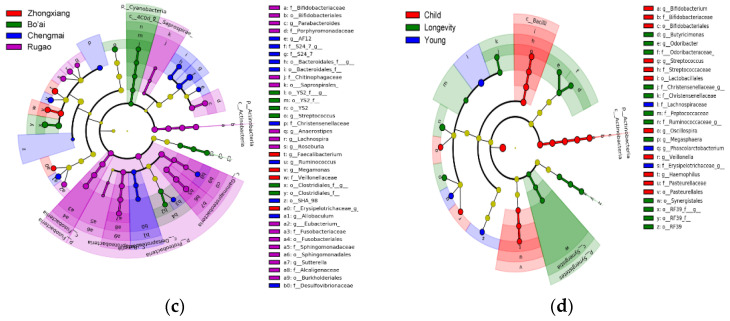
Linear discriminant analysis effect size (LEfSe) analysis of gut microbiota. (**a**,**b**) show the linear discriminant analysis (LDA) score for discriminated genera in the samples grouped by region and age, respectively, (Wilcoxon rank–sum test, α < 0.05, log LDA > 3). (**c**,**d**) are the phylogenetic trees depicting bacterial taxonomic hierarchy that is differentially abundant among different groups.

**Figure 7:**
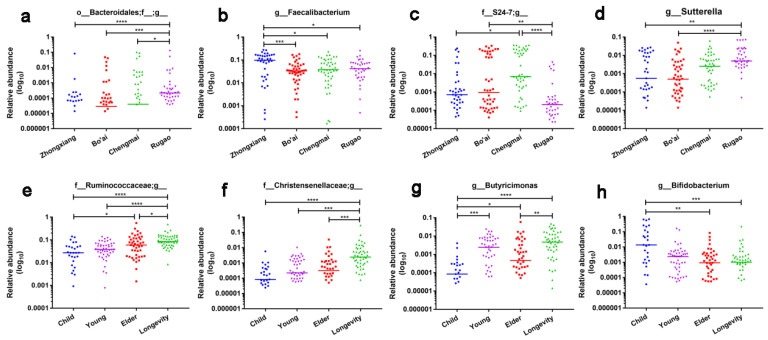
The genera that distribute differently in samples grouped by age (**a**–**d**) and region (**e**–**h**). The colored lines represent the median of each column. * indicates that the difference is significant. * *p* < 0.05, ** *p* < 0.01, *** *p* < 0.001, **** *p* < 0.0001.

All the core genera belonged to *Firmicutes* apart from *Bacteroides*. The results of LEfSe showed that an unclassified *Bacteroidales* genus, *Faecalibacterium*, an unclassified S24-7 genus and *Sutterella* were among the top genera that distinguished the samples by region (Figure 6a,c). The relative abundance of an unclassified *Bacteroidales* genus in the samples from Rugao was significantly higher than that of all the other groups and the relative abundance of *Faecalibacterium* in the subjects from Zhongxiang was significantly higher than that in the other groups (Figure 7a–d). The results also indicated that an unclassified *Ruminococcaceae* genus, an unclassified *Christensenellaceae* genus, *Butyricimonas* and *Bifidobacterium* were among the top genera that distinguished samples grouped by age (Figure 6b,d). The relative abundances of an unclassified *Ruminococcaceae* genus and an unclassified *Christensenellaceae* genus in the Longevity group were significantly higher than that in the other groups (Figure 7e,f). The relative abundance of *Butyricimonas* in Child was significantly lower than that in the other groups, while the relative abundance of *Bifidobacterium* in Child was higher than that in the Young, Elder and Longevity groups, however, the difference between Child and Young was not significant (Figure 7g,h).

### 3.4. Functional Gene Composition of Gut Microbiota Predicted by PICRUSt

**Figure 8:**
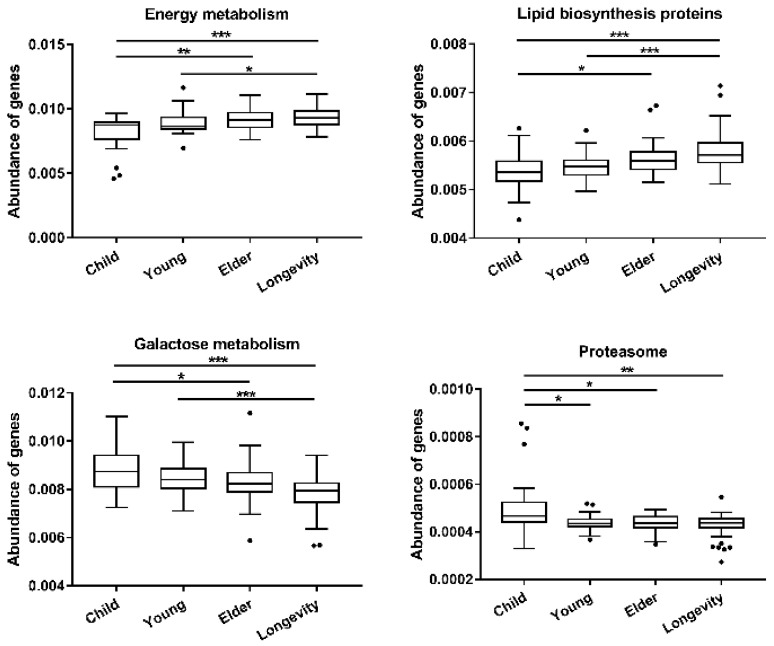
The abundance of several genes among different ages. Boxes represent the interquartile range (IQR) between the first and third quartiles, and the lines inside the boxes represent the median. Whiskers denote the lowest and highest values within 1.5× IQR from the first and third quartiles, respectively. The points represent the values with distance from the median exceeding 1.5× IQR. * indicates that the difference is significant. * *p* < 0.05, ** *p* < 0.01, *** *p* < 0.001.

PICRUSt analysis was used to predict the potential functions of the gut microbiota metagenomes based on 16S rRNA sequences. Analysis of Level 3 KEGG function classes revealed that age exerted significant effects on the functional gene compositions of gut microbiota (*p* < 0.01, PERMANOVA test); however, the functional gene compositions were homogeneous among samples grouped by region (*p* > 0.05, PERMANOVA test). The abundances of genes involved in energy metabolism and lipid biosynthesis revealed an up-regulated tendency from Child to Longevity, while genes involved in galactose metabolism showed a down-regulated tendency; the abundance of proteasome in the Child group was significantly lower than the other groups (Figure 8).

### 3.5. Alpha- and Beta-Diversity Analysis of Bifidobacterium Composition within Gut Microbiota of Subjects from Different Regions and across Age Groups

**Figure 9:**
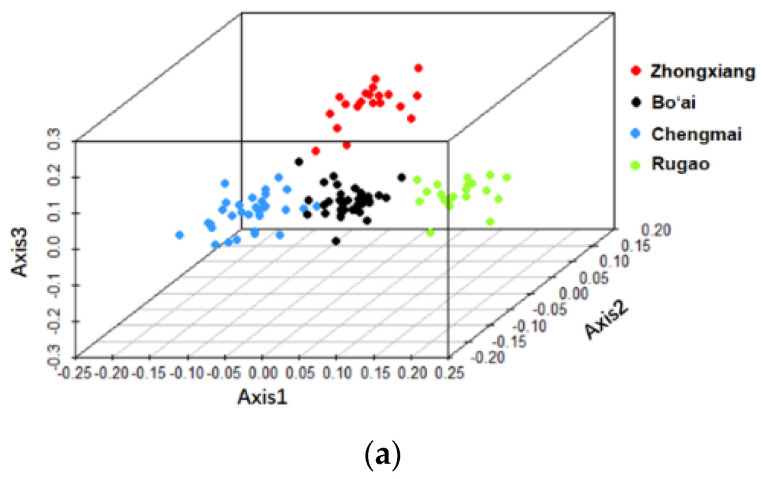

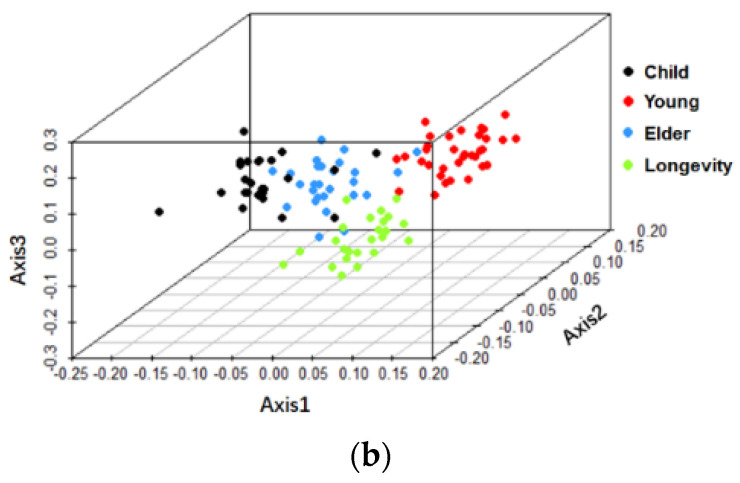
Canonical analysis of principal coordinates (CAP) based on high-throughput sequencing data of the groEL gene of *Bifidobacterium*. (**a**) CAP of *Bifidobacterium* composition of different region groupss. (**b**) CAP of *Bifidobacterium* composition of different age groups.

The analysis of the *Bifidobacterium* composition was performed with high-throughput sequencing based on *Bifidobacterium* specific primers for the groEL gene. Indexes representing alpha-diversity showed that bifidobacterial diversities were similar among different groups with the exception of the Chao1 indexes of the Elder and Longevity groups which were significantly different from each other (Figure S2). The results of PCoA analysis showed that there was no obvious separation of samples grouped by region or age (Figure S3). However, canonical analysis of principal coordinates (CAP) of the sequencing data at OTU level suggested that the composition of *Bifidobacteirum* was different among samples from different regions and of different ages (Figure 9).

### 3.6. Composition of Bifidobacterium in Subjects from Different Regions and Age Groups

**Figure 10:**
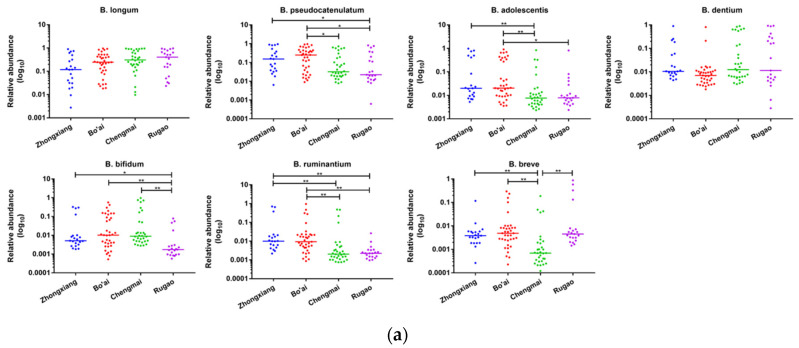

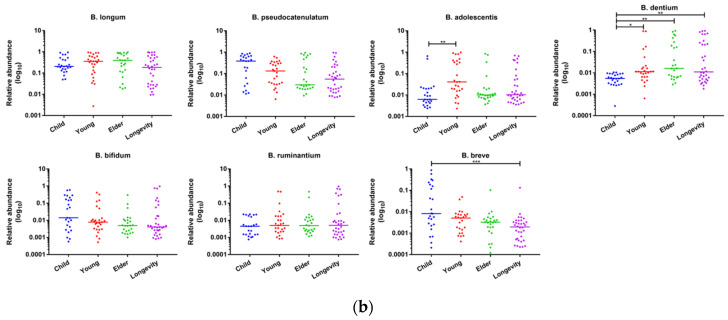
Composition of *Bifidobacterium* in samples from different regions (**a**) and across different ages (**b**). * indicates that the difference is significant. * *p* < 0.05, ** *p* < 0.01, *** *p* < 0.001.

A total of eleven species of *Bifidobacterium* were identified in all samples, in which the relative abundances of seven *Bifidobacterium* species were more than 1%, and *B. longum* was the most abundant species with an average relative abundance of 35.48% among different subjects (Figure S4 and Figure 10). The relative abundance of *B. adolescentis* in the subjects from Bo’ai was significantly higher than that in Chengmai and Rugao; the relative abundance of *B. bifidum* in Rugao was significantly lower than that in the other groups; the relative abundances of *B. ruminantium* in Bo’ai and Zhongxiang were significantly higher than that in Chengmai and Rugao. Additionally, the relative abundance of *B. breve* in Chengmai was significantly lower than other groups. When focusing on the samples by age, Child had a higher relative abundance of *B. pseudocatenulatum* than all the other groups, although the difference was not significant, additionally, Child had significantly lower *B. dentium* than the other groups. By contrast, the relative abundance of *B. adolescentis* in Child was significantly higher than that in Young, and *B. breve* exhibited a significant difference between Child and Longevity, in which the former had a significantly higher abundance.

## 4. Discussion

Various studies have uncovered the effects of several factors (such as age, geography and diet) on the gut microbiota of individuals through high-throughput sequencing. However, few studies have used the same technique to analyze the composition of *Bifidobacterium* in the human gut. Indeed, the composition of *Bifidobacterium* in gut samples has been detected by cultural methods, fluorescence in situ hybridization (FISH), qPCR and denaturing gradient gel electrophoresis (DGGE), which are not appropriate for the analysis of complex samples (such as fecal) and have low resolution power [38,39,40,41]. Therefore, in this study, we analyzed the gut microbiota as well as the composition of *Bifidobacterium* in people from different regions in China (Figure 1) and of different ages (1–5 years, Child; 18–50 years, Young; 60–80 years, Elder; ≥90 years, Longevity), using high-throughput sequencing based on the 16S rRNA gene and the *Bifidobacterium*-specific groEL gene. 

Alpha-diversity (also called within-habitat diversity) presents the species diversity in an area or habitat and reflects the diversity of the composition of microorganisms and is commonly expressed by the Shannon–Wiener index, Simpson index and Chao1 index. Interestingly, the Chao1 index did not differ significantly between the samples grouped by region or age, which indicates that the abundance of microbiota in subjects from different regions or of different ages was similar. The Shannon and Simpson indexes indicated that samples from Bo’ai were rich in microbial species and the distribution of microbes was more uniform compared with samples from Zhongxiang and Rugao. This may be due to the different dietary structure, in which people in Bo’ai predominantly eat wheaten food and vegetables while less meat-based food and fat are consumed. A low-fat diet was reported to associate with higher α-diversity of gut microbiota [42]. The current results revealed that the Shannon and Simpson indexes in Child were significantly lower than that observed in the other groups. Age and geography are known to affect the diversity of the gut microbiota. With regards age, it is generally accepted that the gut microbiota of newborns is acquired from birth onwards where its initial composition is relatively simple but increases in diversity up to 2–5 years of age until it more closely resembles that of adults [43,44,45,46]. Thus the results of our study are in agreement with those of others, in that the microbiota of children is less diverse than that of adults. In terms of geography, analysis of the gut microbiota of healthy children and adults from the Amazonas of Venezuela, rural Malawi and US metropolitan area showed that the abundance of gut bacteria in Americans was significantly lower than that of the other groups [16]. Likewise, canonical analysis showed that samples grouped by region or age were separated from each other, indicating that differences existed in the gut microbiota composition of the different groups. Other factors besides age and geography also impact gut microbiota composition including dietary habits and diseases [47,48,49].

Human gut microbiota mostly consists of obligate anaerobes, in which members of *Firmicutes*, *Bacteroidetes*, *Proteobacteria* and *Actinobacteria* constitute most of the gut microbes [44]. The results in the current study are in line with those of other studies, in that the four dominant phyla accounted for 66.60%, 21.64%, 5.56% and 2.66%, respectively. At the genus level, the eight core genera occupied 28.63% of the whole sequence and the majority of the genera in this core were *Firmicutes,* excluding *Bacteroides* (*Bacteroidetes* phylum). Research on different populations has shown that the human gut microbiota is mainly composed of several genera of *Firmicutes* and *Bacteroidetes* [50]. The most abundant genera in western and Korean subjects were *Bacteroides* and *Faecalibacterium* (*Firmicutes*), respectively, while *Phascolarctobacterium* (*Firmicutes*) was the most abundant in Chinese subjects [50,51,52]. However, in this study, the most abundant genus was *Bacteroides* followed by *Faecalibacterium*. This may be due to the fact that the samples in this study were collected from individuals of Han ethnicity which is the main group in China and does not represent the gut microbiota characterizatics for all the Chinese populations, and a previous study found that the relative abundance of many genera (including *Bacteroides* and *Phascolarctobacterium*) were significantly different among ethnic groups [50]. In addition, *Bacteroides* is positively related with animal protein in the diet, a variety of amino acids and saturated fats, hence, high abundance of *Bacteroides* in the cohort in this study may be associated with a diet rich in animal products [53]. Furthermore, these results confirm that the structure of the gut microbiota is extremely complex and affected by many factors, and it is diverse and individual-specific at genus level.

Gut microbiota composition in the samples from different regions showed that the abundance of *Faecalibacterium* in Zhongxiang was significantly higher than that in other samples. Many studies suggest that *Faecalibacterium* is involved in immuno-regulation. For example, *Faecalibacterium prausnitzii* has been consistently reported as one of the major butyrate producers in the intestine, which could reduce intestinal mucosal inflammation via upregulating PPARγ and inhibiting NF-κB transcription factor activation and interferon gamma (IFN-γ) [54,55,56,57]. Some studies have reported that certain supplemental fermentable fibers in the diet, such as potato fiber, can increase the abundance of *Faecalibacterium* and fecal short chain fatty acid (SCFA) concentrations [58]. The higher abundance of *Faecalibacterium* in samples from Zhongxiang may be due to the specific dietary pattern of this region, which may contain fermentable fibers promoting the proliferation of *Faecalibacterium*, though this needs further investigation.

Ageing is defined as ‘‘the regression of physiological function accompanied by the development of age’’, and the ageing process seriously impacts human gut microbiota profiles [59]. In our study, the relative abundances of *Ruminococcaceae* and *Christensenellaceae* were significantly higher in Longevity compared with all other groups, and the abundance of *Butyricimonas* in Longevity was higher than that in Child and Elder groups. A study profiling the gut microbiota of long-living individuals from Sichuan, China and Italy also showed that OTUs of *Ruminococcaceae* and *Christensenellaceae* were enriched in this age group suggesting that these microorganisms may be beneficial [60]. A higher abundance of *Ruminococcaceae* was observed in subjects supplemented with resistant starch and *Ruminococcaceae* is a well-known butyrate-producer [61,62]. *Christensenellaceae* has been associated with lean body mass index (BMI), indeed, supplementation of *Christensenella* can treat or prevent weight gain, inhibit fat accumulation, reduce excess adiposity and high BMI, and treat or prevent conditions associated with adiposity, such as insulin resistance, metabolic syndrome and diabetes [63,64]. Interestingly, during the recruitment process of this study, almost each longevity volunteer could be described as having a lean body. Previous studies have indicated that increased body size is associated with reducing potential longevity [65]. In the current work, the abundance of *Butyricimonas* in Longevity subjects was significantly higher than that in the Elder group. The increased abundance of *Butyricimonas* has been reported to relate to improved metabolic parameters (including insulin resistance) in mice treated with metformin, which was associated with the downregulation of pro-inflammatory cytokine IL-6 in epididymal fat, and IL-6 levels in adipose tissue reportedly increased with age and attenuated insulin signaling in adipocytes [66,67,68,69]. Ageing is generally accompanied by a chronic low-grade inflammation state (“inflamm-ageing”) which may be linked to the pathogenesis of some chronic diseases, such as cardiovascular disease (CVD) and insulin resistance [70]. Long-living people, especially centenarians, seem to deal with chronic inflammation via anti-inflammatory responses which could be modulated by gut microbes, and this may explain the higher abundance of *Butyricimonas* (butyrate producers with anti-inflammatory effects) in longevities [71,72].

PICRUSt is a computational approach which predicts the functional composition of genes basing on 16S rRNA genes and a reference genome database. In our study, the predicted functional gene compositions were different among groups with different ages. However, our results also showed that functional gene compositions were homogeneous among different region-groups, which was not correlated with the different structure of gut microbiota. Microbial communities often exhibit incredible taxonomic diversity across space, however, this taxonomic diversity may have little effect on the function, because many coexisting but taxonomically distinct microorganisms can encode genes with similar functions (functional redundancy in microbial systems), which could explain the homogeneous compositions of functional genes among different region-groups [73].

*Bifidobacterium* is the most abundant genus in the gut of vaginally-delivered infants after the depletion of oxygen by facultative anaerobes [74], in which *B. breve*, *B. bifidum* and *B. longum* subsp. *infantis* are most prevalent [26,75,76,77]. During adulthood, the abundance of *bifidobacteial* populations decreases to a relatively stable level which decreases again in old age [78]. In this study, the level of *Bifidobacterium* in Child was significantly higher than that in the Elder and Longevity groups (*p* < 0.01). Even though there was no significant difference between the Child and Young groups, the former still had a higher abundance of *Bifidobacterium*. The predominant bifidobacteria in adults are *B. longum* subsp. *longum*, *B. adolescentis* and *B. catenulatum*, and their compositions and abundances in adults are relatively stable [79]. In elderly populations, the abundance and diversity of bifidobacteria significantly decrease and their main bifidobacteria are *B. adolescentis*, *B. longum* subsp. *longum* and *B. angulatum* [80,81,82]. Our current results showed that *B. longum* was the most abundant species in all groups, except Child in which *B. pseudocatenulatum* was most abundant and higher than that in adults even though the difference was not significant, though approached it (*p* = 0.052). A study performed by Wu et al. reported that *B. pseudocatenulatum* was the most dominant *Bifidobacterium* species after dietary intervention with non-digestible but fermentable carbohydrates in a genetically obese child, which suggests that *B. pseudocatenulatum* can be a particular beneficial bacterium given the improvement in bioclinical parameters and weight loss in the study subject [83]. Our results also showed that the relative abundance of *B. dentium* in Child was significantly lower than that in Young. *B. dentium* is usually found in human dental plaque which mostly occurs in adults, thus the lower levels of *B. dentium* in Child may be due to this reason [84]. The relative abundance of *B. adolescentis* in Child was significantly lower than that in Young. Previous studies have shown that *B. adolescentis* is commonly found in adults, and formula-fed babies [85]. In addition, our results indicate that the level of *B. adolescentis* in Chengmai was significantly lower than that in Bo’ai and Zhongxiang, which may be associated with different dietary habits. Indeed, Chengmai is a coastal city where subjects consume diets rich in sea food while the latter two are inland cities where diets contain more starchy foods. A genomic and transcriptomic study of *B. adolescentis* strains revealed a nutrient acquisition strategy targeting starch and starch-like carbohydrates [86], which could explain the different abundances of *B. adolescentis* among volunteers from different regions in this study.

## 5. Conclusions

In summary, by high-throughput sequencing, we analyzed the gut microbiota and *Bifidobacterium* composition of volunteers living in four regions in China with different ages. The results indicate that eight genera are shared by all the samples, but there were also genera that distributed differently in samples grouped by region and age. *Faecalibacterium* was enriched in samples from Zhongxiang; *Ruminococcaceae* and *Christensenellaceae* were enriched in the Longevity group; *Bifidobacterium* was enriched in the Child group. The detection of *Bifidobacterium* indicated that *B. longum* was the most abundant *Bifidobacterium* in almost all samples except for Child, in which *B. pseudocatenulatum* was most abundant. In addition, the results show that the abundance of *B. adolescentis* and *B. dentium* were lower in Child. Further studies are needed to investigate if causal relationships exist between the varying microbes and certain populations, and how these gut microorganisms interact with each other and with their host, and how they affect health and the aging process, as well as the intrinsic mechanisms involved. 

## Supplementary Materials

The following are available online at https://www.mdpi.com/2076-2607/8/8/1108/s1: Figure S1: Principal coordinates analysis (PCoA) of weighted UniFrac distances based on the high-throughput sequencing data of the V4 region of the 16S rRNA gene.; Figure S2: Alpha-diversities of the bifidobacterial communities within subjects from different regions and across age groups. Boxes show the interquartile range (IQR) between the first and third quartiles, and the lines inside boxes represent the median. Whiskers denote the lowest and highest values within 1.5×IQR from the first and third quartiles, respectively. The points present the values with distance from the median exceeding 1.5 times IQR.; Figure S3: Figure. S3 Principal coordinates analysis (PCoA) of weighted UniFrac distances based on high-throughput sequencing of the *groEL* gene.; Figure S4: Composition of the bifidobacterial community within subjects from different regions (a) and across different ages (b).; Table S1: Basic information of experimental subjects.; Table S2: *p*-value of PERMANOVA test between each region-group., Table S3: *p*-value of PERMANOVA test between each age-group.
