# Supplementary material for: Diversity of Gut Microbiota and Bifidobacterial Community of Chinese Subjects of Different Ages and from Different Regions
Source: Microorganisms. 2020 Jul 24;8(8):1108. doi: 10.3390/microorganisms8081108 (PMC7464982; doi:10.3390/microorganisms8081108)
Supplement: Supplementary file 1 [file microorganisms-08-01108-s001.pdf]

Supplementary Materials

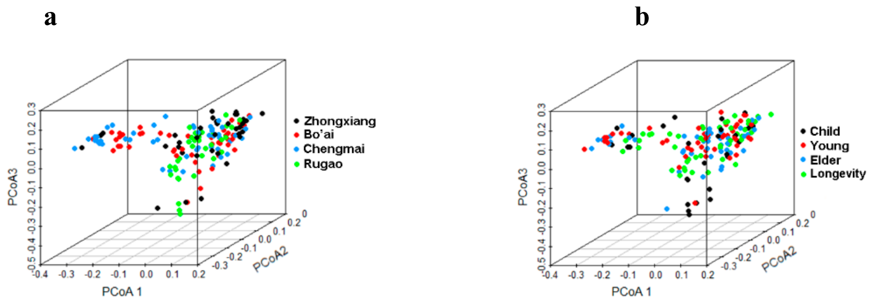

Figure. S1 Principal coordinates analysis (PCoA) of weighted UniFrac distances based on the high-throughput sequencing data of the V4 region of the 16S rRNA gene.

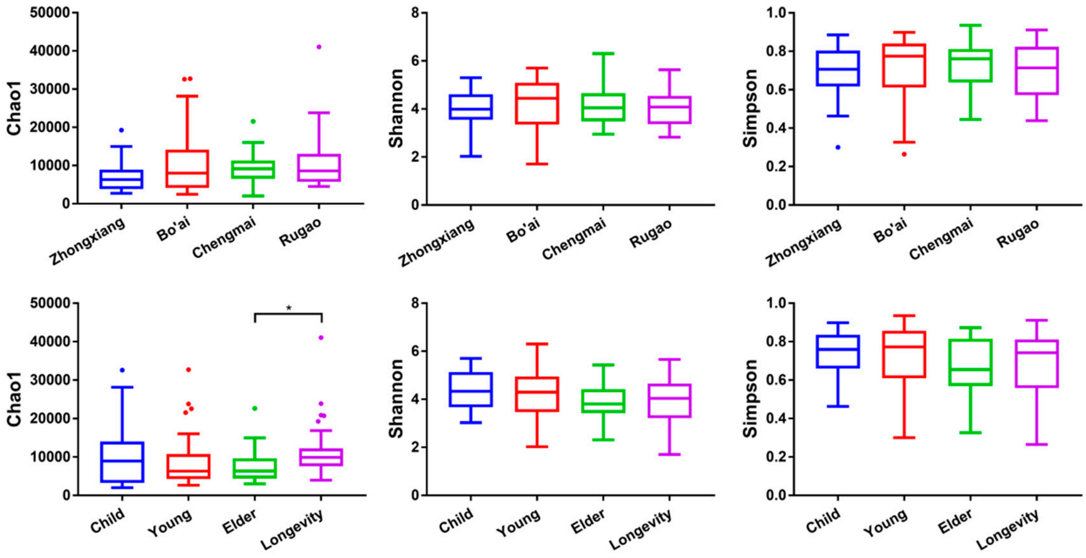

Figure. S2 Alpha-diversities of the bifidobacterial communities within subjects from different regions and across age groups. Boxes show the interquartile range (IQR) between the first and third quartiles, and the lines inside boxes represent the median. Whiskers denote the lowest and highest values within 1.5×IQR from the first and third quartiles, respectively. The points present the values with distance from the median exceeding 1.5 times IQR.

a b

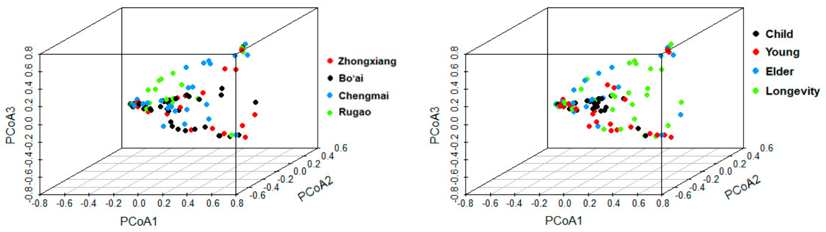

Figure. S3 Principal coordinates analysis (PCoA) of weighted UniFrac distances based on high-throughput sequencing of the groEL gene.

a

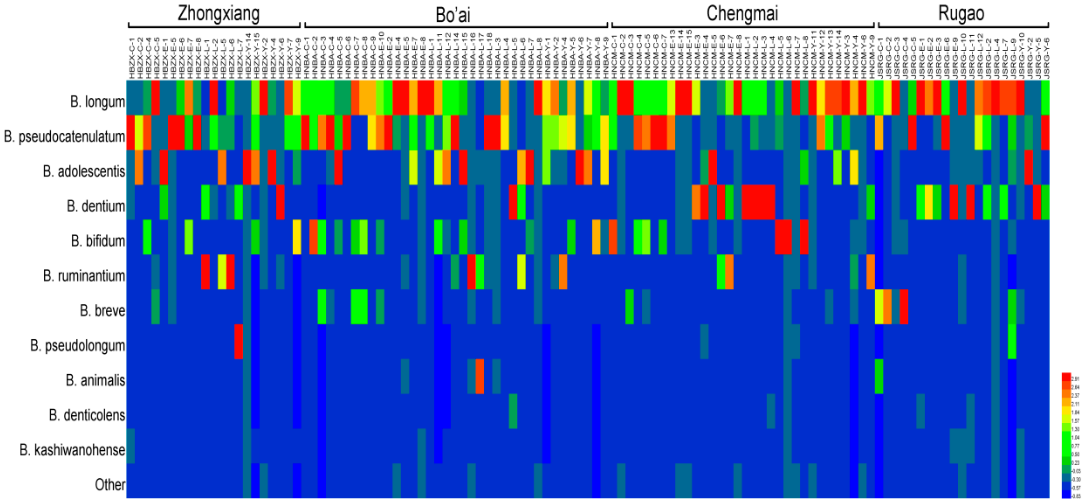

b

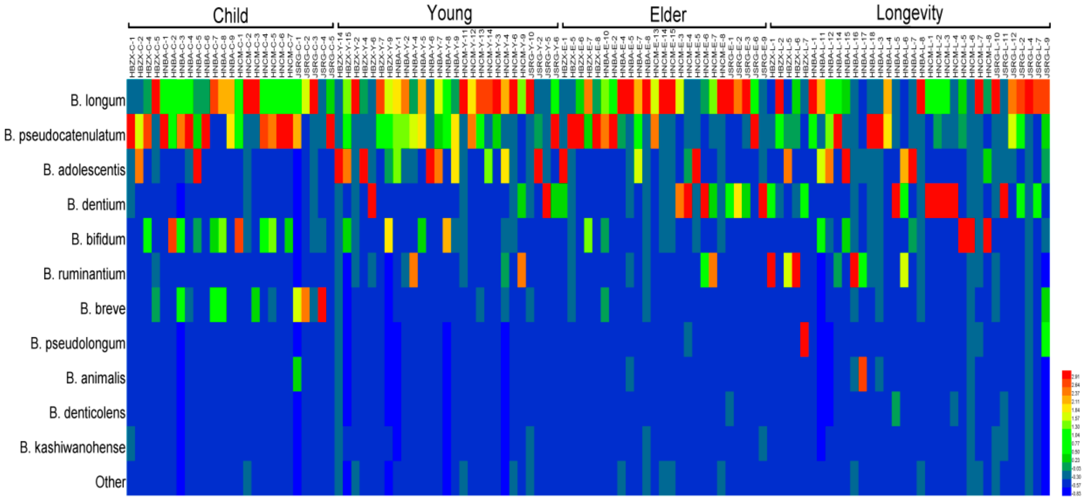

Figure. S4 Composition of the bifidobacterial community within subjects from different regions (a) and across different ages (b).

Table S1 Basic information of experimental subjects.

Sample ID	Sampling Region	Age (years)	Gender	Ethnicity	Region Group	Age Group
HBZX-C-1	Zhongxiang, Hubei	1	Male	Han	Zhongxiang	Child
HBZX-C-2	Zhongxiang, Hubei	5	Male	Han	Zhongxiang	Child
HBZX-C-4	Zhongxiang, Hubei	2	Male	Han	Zhongxiang	Child
HBZX-C-5	Zhongxiang, Hubei	3	Female	Han	Zhongxiang	Child
HBZX-Y-14	Zhongxiang, Hubei	27	Male	Han	Zhongxiang	Young
HBZX-Y-15	Zhongxiang, Hubei	24	Male	Han	Zhongxiang	Young
HBZX-Y-2	Zhongxiang, Hubei	42	Male	Han	Zhongxiang	Young
HBZX-Y-4	Zhongxiang, Hubei	49	Male	Han	Zhongxiang	Young
HBZX-Y-6	Zhongxiang, Hubei	47	Male	Han	Zhongxiang	Young
HBZX-Y-7	Zhongxiang, Hubei	46	Female	Han	Zhongxiang	Young
HBZX-Y-9	Zhongxiang, Hubei	40	Female	Han	Zhongxiang	Young
HBZX-E-1	Zhongxiang, Hubei	77	Female	Han	Zhongxiang	Elder
HBZX-E-5	Zhongxiang, Hubei	65	Male	Han	Zhongxiang	Elder
HBZX-E-6	Zhongxiang, Hubei	64	Male	Han	Zhongxiang	Elder
HBZX-E-7	Zhongxiang, Hubei	62	Female	Han	Zhongxiang	Elder
HBZX-E-8	Zhongxiang, Hubei	60	Female	Han	Zhongxiang	Elder
HBZX-L-1	Zhongxiang, Hubei	105	Male	Han	Zhongxiang	Longevity
HBZX-L-2	Zhongxiang, Hubei	103	Male	Han	Zhongxiang	Longevity
HBZX-L-5	Zhongxiang, Hubei	95	Male	Han	Zhongxiang	Longevity
HBZX-L-6	Zhongxiang, Hubei	95	Male	Han	Zhongxiang	Longevity
HBZX-L-7	Zhongxiang, Hubei	90	Male	Han	Zhongxiang	Longevity
HNBA-C-1	Bo'ai, Henan	5	Male	Han	Bo'ai	Child
HNBA-C-2	Bo'ai, Henan	4	Male	Han	Bo'ai	Child
HNBA-C-3	Bo'ai, Henan	3	Female	Han	Bo'ai	Child
HNBA-C-4	Bo'ai, Henan	3	Female	Han	Bo'ai	Child
HNBA-C-5	Bo'ai, Henan	2	Male	Han	Bo'ai	Child
HNBA-C-6	Bo'ai, Henan	2	Male	Han	Bo'ai	Child
HNBA-C-7	Bo'ai, Henan	1	Female	Han	Bo'ai	Child
HNBA-C-8	Bo'ai, Henan	1	Female	Han	Bo'ai	Child
HNBA-C-9	Bo'ai, Henan	1	Female	Han	Bo'ai	Child
HNBA-Y-1	Bo'ai, Henan	48	Male	Han	Bo'ai	Young
HNBA-Y-2	Bo'ai, Henan	46	Female	Han	Bo'ai	Young
HNBA-Y-4	Bo'ai, Henan	44	Male	Han	Bo'ai	Young
HNBA-Y-5	Bo'ai, Henan	43	Female	Han	Bo'ai	Young
HNBA-Y-6	Bo'ai, Henan	41	Male	Han	Bo'ai	Young
HNBA-Y-7	Bo'ai, Henan	41	Female	Han	Bo'ai	Young
HNBA-Y-8	Bo'ai, Henan	38	Male	Han	Bo'ai	Young
HNBA-Y-9	Bo'ai, Henan	36	Female	Han	Bo'ai	Young
HNBA-E-10	Bo'ai, Henan	65	Female	Han	Bo'ai	Elder
HNBA-E-2	Bo'ai, Henan	77	Female	Han	Bo'ai	Elder
HNBA-E-4	Bo'ai, Henan	74	Male	Han	Bo'ai	Elder

HNBA-E-5	Bo'ai, Henan	73	Male	Han	Bo'ai	Elder
HNBA-E-7	Bo'ai, Henan	71	Male	Han	Bo'ai	Elder
HNBA-E-8	Bo'ai, Henan	66	Female	Han	Bo'ai	Elder
HNBA-L-1	Bo'ai, Henan	108	Female	Han	Bo'ai	Longevity
HNBA-L-11	Bo'ai, Henan	92	Male	Han	Bo'ai	Longevity
HNBA-L-12	Bo'ai, Henan	91	Female	Han	Bo'ai	Longevity
HNBA-L-14	Bo'ai, Henan	90	Male	Han	Bo'ai	Longevity
HNBA-L-15	Bo'ai, Henan	90	Male	Han	Bo'ai	Longevity
HNBA-L-16	Bo'ai, Henan	90	Female	Han	Bo'ai	Longevity
HNBA-L-17	Bo'ai, Henan	90	Female	Han	Bo'ai	Longevity
HNBA-L-18	Bo'ai, Henan	92	Female	Han	Bo'ai	Longevity
HNBA-L-3	Bo'ai, Henan	95	Female	Han	Bo'ai	Longevity
HNBA-L-4	Bo'ai, Henan	95	Female	Han	Bo'ai	Longevity
HNBA-L-5	Bo'ai, Henan	95	Female	Han	Bo'ai	Longevity
HNBA-L-6	Bo'ai, Henan	93	Male	Han	Bo'ai	Longevity
HNBA-L-7	Bo'ai, Henan	93	Female	Han	Bo'ai	Longevity
HNBA-L-8	Bo'ai, Henan	93	Female	Han	Bo'ai	Longevity
HNCM-C-1	Chengmai, Hainan	3	Male	Han	Chengmai	Child
HNCM-C-2	Chengmai, Hainan	5	Male	Han	Chengmai	Child
HNCM-C-3	Chengmai, Hainan	4	Male	Han	Chengmai	Child
HNCM-C-4	Chengmai, Hainan	5	Male	Han	Chengmai	Child
HNCM-C-5	Chengmai, Hainan	4	Male	Han	Chengmai	Child
HNCM-C-6	Chengmai, Hainan	3	Male	Han	Chengmai	Child
HNCM-C-7	Chengmai, Hainan	3	Male	Han	Chengmai	Child
HNCM-Y-11	Chengmai, Hainan	30	Male	Han	Chengmai	Young
HNCM-Y-12	Chengmai, Hainan	30	Male	Han	Chengmai	Young
HNCM-Y-13	Chengmai, Hainan	27	Male	Han	Chengmai	Young
HNCM-Y-14	Chengmai, Hainan	24	Male	Han	Chengmai	Young
HNCM-Y-3	Chengmai, Hainan	43	Female	Han	Chengmai	Young
HNCM-Y-4	Chengmai, Hainan	41	Male	Han	Chengmai	Young
HNCM-Y-6	Chengmai, Hainan	49	Male	Han	Chengmai	Young
HNCM-Y-9	Chengmai, Hainan	31	Male	Han	Chengmai	Young
HNCM-E-13	Chengmai, Hainan	60	Male	Han	Chengmai	Elder
HNCM-E-14	Chengmai, Hainan	62	Female	Han	Chengmai	Elder
HNCM-E-15	Chengmai, Hainan	65	Male	Han	Chengmai	Elder
HNCM-E-3	Chengmai, Hainan	73	Male	Han	Chengmai	Elder
HNCM-E-4	Chengmai, Hainan	73	Female	Han	Chengmai	Elder
HNCM-E-5	Chengmai, Hainan	80	Female	Han	Chengmai	Elder
HNCM-E-6	Chengmai, Hainan	79	Female	Han	Chengmai	Elder
HNCM-E-7	Chengmai, Hainan	78	Male	Han	Chengmai	Elder
HNCM-E-8	Chengmai, Hainan	78	Male	Han	Chengmai	Elder
HNCM-L-1	Chengmai, Hainan	105	Female	Han	Chengmai	Longevity
HNCM-L-2	Chengmai, Hainan	105	Male	Han	Chengmai	Longevity
HNCM-L-3	Chengmai, Hainan	105	Male	Han	Chengmai	Longevity

HNCM-L-4	Chengmai, Hainan	98	Male	Han	Chengmai	Longevity
HNCM-L-5	Chengmai, Hainan	96	Female	Han	Chengmai	Longevity
HNCM-L-6	Chengmai, Hainan	95	Female	Han	Chengmai	Longevity
HNCM-L-7	Chengmai, Hainan	94	Female	Han	Chengmai	Longevity
HNCM-L-8	Chengmai, Hainan	93	Female	Han	Chengmai	Longevity
JSRG-C-1	Rugao, Jiangsu	1	Female	Han	Rugao	Child
JSRG-C-2	Rugao, Jiangsu	4	Female	Han	Rugao	Child
JSRG-C-3	Rugao, Jiangsu	2	Male	Han	Rugao	Child
JSRG-C-4	Rugao, Jiangsu	2	Female	Han	Rugao	Child
JSRG-C-5	Rugao, Jiangsu	4	Male	Han	Rugao	Child
JSRG-Y-10	Rugao, Jiangsu	50	Female	Han	Rugao	Young
JSRG-Y-2	Rugao, Jiangsu	33	Female	Han	Rugao	Young
JSRG-Y-5	Rugao, Jiangsu	42	Female	Han	Rugao	Young
JSRG-Y-6	Rugao, Jiangsu	41	Female	Han	Rugao	Young
JSRG-E-1	Rugao, Jiangsu	67	Male	Han	Rugao	Elder
JSRG-E-2	Rugao, Jiangsu	67	Female	Han	Rugao	Elder
JSRG-E-3	Rugao, Jiangsu	75	Female	Han	Rugao	Elder
JSRG-E-6	Rugao, Jiangsu	67	Female	Han	Rugao	Elder
JSRG-E-9	Rugao, Jiangsu	64	Male	Han	Rugao	Elder
JSRG-L-10	Rugao, Jiangsu	95	Female	Han	Rugao	Longevity
JSRG-L-11	Rugao, Jiangsu	96	Female	Han	Rugao	Longevity
JSRG-L-12	Rugao, Jiangsu	95	Female	Han	Rugao	Longevity
JSRG-L-2	Rugao, Jiangsu	95	Female	Han	Rugao	Longevity
JSRG-L-4	Rugao, Jiangsu	92	Male	Han	Rugao	Longevity
JSRG-L-7	Rugao, Jiangsu	102	Female	Han	Rugao	Longevity
JSRG-L-9	Rugao, Jiangsu	96	Male	Han	Rugao	Longevity

Table S2 *P*-value of PERMANOVA test between each region-group

	Bo'ai	Chengmai	Rugao
Zhongxiang	0.002	0.001	0.001
Bo'ai		0.028	0.001
Chengmai			0.001

Table S3 *P*-value of PERMANOVA test between each age-group

	Young	Elder	Longevity
Child	0.004	0.022	0.001
Young		0.258	0.003
Elder			0.025
